# Freshness Quality and Shelf Life Evaluation of the Seaweed *Ulva rigida* through Physical, Chemical, Microbiological, and Sensory Methods

**DOI:** 10.3390/foods10010181

**Published:** 2021-01-18

**Authors:** Fini Sánchez-García, Ignacio Hernández, Víctor M. Palacios, Ana M. Roldán

**Affiliations:** 1 Department of Chemical Engineering and Food Technology, Faculty of Sciences, University of Cádiz, Puerto Real, 11510 Cádiz, Spain; victor.palacios@uca.es (V.M.P.); ana.roldan@uca.es (A.M.R.); 2 Department of Biology, Division of Ecology, University of Cádiz, Puerto Real, 11510 Cádiz, Spain; ignacio.hernandez@uca.es

**Keywords:** *Ulva rigida*, seaweeds, freshness, quality, shelf life, storage temperature

## Abstract

In Europe, the consumption of seaweeds and derived products has increased in recent years, due to the expansion of Asian cuisine and the emergence of many top-level chefs. Often in collaboration with scientists, many have initiated a new gastronomy using algae. However, little is known about the quality and degree of freshness of seaweeds for direct consumption or fresh use. For this reason, different analytical methods were applied to test sea vegetables and other marine products. These methods included physical (a_w_, pH, color, and texture), chemical (total volatile base nitrogen, TVB-N; and trimethylamine, TMA-N) parameters, microbiological count, and sensory evaluation. In this study, freshness quality and shelf life of the green seaweed *Ulva rigida* (UR) was evaluated during a 12-day period, stored at 4 and 16 °C. The parameters that proved to be most useful for evaluating its freshness were the TVB, TMA, microbiological, and sensory analyses. The physicochemical and microbiological parameters established a shelf life of UR of 6 days for a storage temperature of 16 °C and up to 10 days for a storage temperature of 4 °C. The changes that UR undergoes during its storage from the sensory point of view are more pronounced than those produced from the physicochemical point of view, which can condition its applications.

## 1. Introduction

The interest in healthier diets containing marine products and their ingredients, such as oils and fish proteins, macro- and microalgae and their bioactive compounds, has increased in recent years. Seaweeds are rich in several nutrients [1] and bioactive compounds [2], with attractive technological properties [3] and other potential health benefits [4]. Furthermore, seaweeds have interesting organoleptic properties, such as aromas (marine, crustacean, and green notes) and flavors (umami). These properties show the potential of seaweeds to be included in processed food, and for the development of new marine foods [5] and culinary innovations [6] with great gastronomic value [7,8]. As a consequence, new types of seaweed industries have developed in recent years to meet consumers’ demands for these products [9]. For example, the interest in seaweeds has focused on Ulvales as a novel food product in Western countries [6,7]. Sea lettuce (*Ulva* spp.) has an interesting nutritional composition (18% protein, 3.5% fat, 45% carbohydrates, and approximately 31% fiber of dry weight) [8,10,11].

Nowadays, seaweeds are sold in different forms, such as raw products (dried or salted), condiments, and mashed algae. However, seaweeds have a perishable nature similar to other seafoods. For this reason, they must be immediately subjected to drying processes to reduce water activity and increase the shelf life [12]. Drying on nets [13], or industrial air drying at 50 °C [12,14], are the most common processes used to dry seaweeds and they noticeably influence seaweeds’ functional and sensory properties [12,15].

Quality of fresh seaweeds and their shelf life assessment have not been studied in depth, but it is necessary to assure the minimum quality of the product and especially its food safety. The acceptance on the seafood market depends on the state of freshness (mainly appearance and smell). In the case of fish and other seafoods, the main quality parameters for freshness are aroma, flavor, texture, and sensory response [16]. Conventional methods including sensory (quality index methods, QIMs), physical (color, texture, and use of sensors for specific properties), microbiological (viable microorganism count) and chemical methods (biogenic amines, total volatile bases (TVBs), trimethylamine (TMA), nucleotides, and nucleosides) [17,18,19] are used to evaluated freshness and quality of marine products.

Sensory analysis has been considered the single tool to evaluate freshness in seaweeds, but some authors also studied color, texture, moisture loss, soluble proteins, and microbiological count as quality parameters of brown and red seaweeds subjected to different heat treatments or storage temperatures [20,21]. However, not all parameters were useful for determining quality changes and needed adjustments according to the type of seaweed. Therefore, it is necessary to establish suitable methods for detecting seaweed spoilage and determine its stage of freshness, quality, and shelf life depending on the seaweed variety and the postharvest storage conditions. These methods should establish not only the shelf life and freshness of seaweeds, but also improve the preservation techniques.

The aim of this study was to evaluate chemical, physical, and sensory changes in the green seaweed *Ulva rigida* during its spoiling at different temperatures of storage, to define the parameters of freshness, quality, and shelf life. For this evaluation, different methods were used, such as sensory, physical (a_w_, pH, color, and texture), chemical (TVB and TMA), and microbiological analysis, previously applied to seaweeds or other marine products (fish and shellfish).

## 2. Materials and Methods

### 2.1. Collection and Preparation of Samples

A total amount of approximately 3 kg of fresh green seaweed *Ulva rigida* (UR) was harvested from earthen ponds in February 2018 (San Fernando, Cádiz, Spain; 36°29′11.9″ N, 6°9′26.23″ W). Samples were immediately carried to the laboratory in refrigerated conditions (4 °C). Subsequently, the samples were maintained for 24 h in sterile seawater to remove sand remains and epiphytes. Then, samples were drained, packed in 12 polyethylene minigrip (ziplock) bags of 200 g each, and stored at two different temperatures, for 12 days in dark conditions. 4 °C (stored in a refrigerator), and 16 °C (stored in an air-jacket incubator), were selected as storage temperatures according to previous studies of vegetable preservations [22,23], fish [24,25] and other seafood [26] within a range between 2 and 20 °C. Samples were taken from each bag on days 2, 4, 6, 8, 10, and 12. For day 0, as a starting point (zero time), samples were taken directly from the washed and drained pool of seaweed.

Seaweed samples, taken in duplicate, were immediately analyzed using microbiological and sensory methods. Physical parameters were also determined (a_w_, pH, texture, and color). The rest was frozen at −20 °C until the assay of chemical parameters was carried out (TVB, total volatile bases; and TMA, trimethylamine).

### 2.2. Physical Analysis

Water activity (a_w_) was measured using a hygrometer (Aqualab Serie 3, Decagon Devices, Pullman, WA, USA). The equipment was calibrated with standard salt solutions and internal temperature control to measure water activity at 25 °C (±0.2 °C). Samples of UR (approximately 0.5 g) were placed in measurement capsules (Decagon Devices Inc., Pullman, WA, USA), ensuring the complete cover of the capsules’ base. The analysis was performed in triplicate.

pH values were determined with a digital pH-meter (Micro pH 2001, Crison Instruments, Barcelona, Spain), equipped with a probe 52-32 (Crison Instruments, Barcelona, Spain). Values were recorded by dipping the pH-meter probe into 20 g seaweed homogenate. pH values were calculated as the mean of the triplicates. The pH-meter was calibrated every 10 measurements using buffers at pH 4.0 and 7.0.

Drip loss was defined by Nayyar and Skonberg [21] as the cellular fluids lost during storage. The drip water loss was calculated as percent water lost compared to the initial sample weight.

Seaweed color during storage was assessed using a portable spectrophotometer (CM-2600d, Konica Minolta Co., Osaka, Japan). The spectrophotometer was calibrated with standard white and black plates. The SpectraMagicTM NX software was used to record measurements and analyze the color. The samples (~0.5 g) were uniformly placed to completely cover the surface of a Petri dish. Luminosity (L*), redness (a*), and yellowness (b*) values were measured five times for each sample after Petri dish rotation. Color change (∆*E*) over time was calculated on each sampling day in comparison to day 0, using the Silva and Silva [27] formula:

(1) $$\Delta E=\sqrt{{\left(L_{0}-L\right)}^{2}+{\left(a_{0}-a\right)}^{2}+\;{\left(b_{0}-b\right)}^{2}}$$

where *L*_0_, *a*_0_ and *b*_0_ are the lightness, redness, and yellowness values for day 0 of testing. 

The texture profile was analyzed using a texture analyzer (TA1, Lloyd-instruments, AMETEK GmbH, Meerbusch, Germany) platform equipped with an 80 N compression load cell. The seaweed was cut into (3 cm × 3 cm) square pieces and kept at room temperature for the texture analysis. A flat-bottomed cylindrical probe Type P/0.5 of 12.5 mm diameter was used to compress the samples with 4 mm s^−1^ test speed. Seaweed cohesiveness, crispness, hardness, and penetration work were recorded by the texture analysis software Nexygen Plus 3.0 for Windows (Lloyd-instruments, AMETEK GmbH, Meerbusch, Germany). Three replicates for each sample of temperature and day were assessed.

### 2.3. Chemical Analysis

The content of total volatile base nitrogen (TVB-N) in UR samples were determined using Conway’s dish method according to the procedure of Cobb et al. [28] with some specifications. The extract was prepared by mixing 2 g of minced seaweed with 8 mL of 4% TCA in a 50 mL screw cap bottle and homogenized. It was stirred for five minutes at room temperature using an ultrasound machine. Subsequently, the extract was filtered through Whatman N°1 filter papers and the filtered solution was kept in a glass tube. A Conway unit for each sample and another for each blank were used. 1 mL of 1% boric acid solution was added to the inner ring of each unit. Then, 1 mL of the sample extract and 1 mL of saturated potassium carbonate solution were carefully added to the outer ring of each unit, and the units were immediately covered and closed with a clip. The units were incubated at 45 °C for 45 min. The inner ring solution was then titrated with 0.02 N HCl until the green colored solution turned pink. An average titrate volume of HCl was determined from the results of three titrations for each seaweed sample. A blank test was also carried out using 1 mL of 1% TCA, instead of sample extract. TVB-N content was then determined and expressed as mg 100 g^−1^. The same protocol was applied to assess the trimethylamine (TMA-N) content of the seaweed, with a difference prior, the addition of potassium carbonate. 1 mL of 10% neutralized formalin was added into the inner ring, to interact with the ammonia, only allowing the TMA-N to diffuse over the Conway dish. The concentration was also expressed as mg 100 g^−1^.

### 2.4. Microbiological Analysis

The microbial cell count with microscopic examination of the exudate liquid from stored samples was proposed as an easy and quick approximation to assess the quantity of microorganisms involved in freshness loss in UR [29]. Therefore, aliquots of exudate water were taken from each bag, and microbial cells were counted in serial dilutions. Samples (1 mL) were stained with methylene blue (1% *v/v*) to increase the contrast of microbial cells [30]. The count was performed with an optical microscope (Leica Microsystem GmbH, Wetzlar, Germany) using cell counting chambers (Improved Neubauer Chamber, Brand GmbH and Co. KG., Wertheim, Germany). Counts were assessed in duplicate and expressed as number of microbial cells per mL.

### 2.5. Sensory Analysis

The physical and biochemical analyses described above were complemented with sensory analysis. A total of 10 trained panelists, identified as familiar with sea products and sensory analysis, took part in the analysis. Each panelist assessed two UR samples from the two temperatures in a single session. A total of seven sessions were conducted. 

Prior to the analysis, a descriptor-development session was carried out to determine the appropriate descriptors that could describe seaweeds according to Regulation UNE-ISO 13299:2016 [31]. The panel leader chose the descriptors used for the training based on those used in the sensory analysis of seaweeds and related products [32,33] and consensus was found among the panelists. In total, 32 descriptors were chosen for the final evaluation (see Supplementary file).

The selected descriptors for sensory evaluation included attributes for appearance, aroma, flavor, tactile mouthfeel, taste, and touch. Three characteristics were appearance-related attributes (general aspect, color intensity, brightness); nine were fish and sea-related attributes (fresh fish, cooked fish, dry/salty fish, rotten fish, seaweed, mollusk, crustacean, coast/rock, sludge); six were vegetal-related attributes (fresh grass, raw vegetable, cooked vegetable, fruit, fungi/mold); three were other aromas (spices, urine, strong, rotten odor); five descriptors were tactile mouthfeel and taste-related attributes (fishy, vegetable, flavor intensity, persistence, chewiness); and three were touch-related attributes (hardness, stickiness, and elasticity). The intensity of each attribute was measured on a lineal, non-structured scale from 0 (sensation not perceived) to 5 (maximum sensation). Each sample of UR was presented in crystal cups covered with a watch glass and held at room temperature for 20 min before sensory analysis.

### 2.6. Statistical Analysis

A one-way ANOVA was applied to evaluate the effect of time on seaweed freshness parameters. The Tukey’s post hoc test was used to identify significant differences among sampling points. Differences between preservation temperatures at each sampling point were determined by Student’s *t*-tests. Normality and homogeneity of variances were previously confirmed using the Kolmogorov-Smirnov´s and Levene’s tests, respectively. Statistical significance was accepted at *p* < 0.05. Data were analyzed using the GraphPad Prism software, version 6.01 for Windows (San Diego, CA, USA).

## 3. Results and Discussion

### 3.1. Effects of Storage at Different Temperatures on the Physical Properties of Ulva rigida

The a_w_, pH, drip loss, and color parameters are shown in Table 1. The a_w_, is a parameter widely used by the food industry to establish the stability, mainly microbiological, of food and to determine the best methods of preservation [34]. The initial value of UR (0.957 ± 0.002) was below the value presented by some marine products (0.99–0.97), vegetables (0.97–0.98), and others seaweeds (0.978–0.989) [35], although not enough to inhibit bacterial growth. During storage, there were small variations in the a_w_ value, with a tendency to decrease (Table 1). This indicated a slight loss of humidity and desiccation reflected in the percentage of water per exudate (Table 1), which reached 7 and 9% in UR stored at 4 and 16 °C, respectively. However, the evolution of a_w_ did not occur in the same way at both temperatures. At 4 °C it was progressive, and at 16 °C this decrease occurred during the first days, with its day 4 value equal to the day 10 value of the samples stored at 4 °C. Afterwards, the a_w_ value remained the same, indicating that it was influenced by the saturation humidity accumulated in the bag.

The pH is a physical control method widely used in marine products indicative of enzymatic or microbial activity. As shown in Table 1, UR starts from a pH of 6.67, a value very similar to other marine species such as fish [36], mollusks [37], and shellfish [38] and even other edible algae species preserved through high-pressure processing [35]. This value indicates that seaweeds are species with a pH close to neutral and, therefore, vulnerable to microorganisms. An increase in pH can cause an accelerated growth of some microorganisms and, in turn, produce an increase in pH. The results showed how UR stored at 4 °C suffered a slight increase in pH during the first few days. Afterwards, a decrease was observed, until the tenth day when it increased strongly. This effect probably took place through the formation and accumulation of acids, such as lactic acid, as occurs in other marine species.

In the case of mollusks, especially bivalves, it has been shown that they suffer the usual alterations of microbiological origin as a result of the fermentation of carbohydrates, split to form mainly lactic acid and alcohol, under anaerobic conditions [39].

In addition, a similar evolution has been observed in some vegetables, albeit due to the formation of acids during ripening from ethylene biosynthesis, such as 1-aminocyclopropane-1-carboxylic acid (ACC) and indolacetic acid (IAA) [40,41]. In the last few days, the pH tends to increase, a similar evolution to that observed in fish once the signs of alteration and deterioration begin [36] or in other marine products, such as sea cucumber [42]. This is a consequence of the formation of basic volatile compounds from protein degradation [43]. Seaweeds stored at 16 °C showed a different evolution of pH, producing a progressive increase of pH until the eighth day, most likely as a result of the generation of basic compounds [42,44]. Finally, a decrease was observed due to the formation of biogenic amines from proteins, and favoring the growth of acetic and lactic bacteria.

The loss of water by exudate release (drip loss), seems to be a parameter indicative of the deterioration of UR and loss of quality. Drip loss (Table 1) was observed at both storage temperatures and their evolution was very similar. As in vegetables, cold damage causes breakage of the cellular structure and, as a consequence, loss of cellular liquid. This phenomenon is also observed in seaweed, although the greater or lesser loss by exudate seems to be related to the species [21]. In the present study, the determined water loss was higher than the values obtained by Nayyar and Skonberg [21] for *Gracilaria tikvahiae* (2–4.5%), being higher at 16 °C. This phenomenon could be explained by the thin and flat structure of *U. rigida*, very similar to green leafy vegetables, such as lettuce, whose increased respiratory activity accelerates water loss [45].

**Figure 1:**
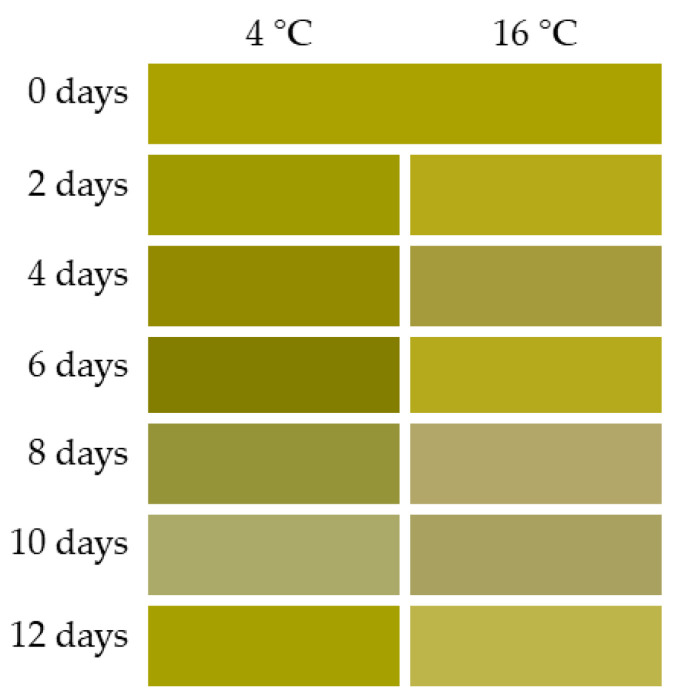
Color obtained with CM-2600d portable spectrophotometer at both temperatures over time, represented by days.

Color is one of the most important factors for consumers, marking the preference and acceptability of the products [46]. Therefore, a loss of the initial color lead to a decrease in quality. The green and luminous color characteristic of UR evolved during storage, with more pronounced changes at 16 °C, as shown in Table 1. At 4 °C, the main change was observed in the value of b* and L*, decreasing during the first days of storage, while a* remained practically constant. However, at 16 °C, there was an increase in the a* value and a decrease in the b* value, while L* did not vary significantly during the storage period. The color change (Δ*E*) values for samples stored at this temperature increased significantly over time and faster than when stored at 4 °C (Table 1). This behavior indicates a clear effect of temperature on color. The green color of UR (Figure 1) changed progressively as a result of the decrease of b*, corresponding to the yellow color. At 16 °C, there was a faster increase in the green color associated with the breakage of the fucoxanthin pigment [20]. Subsequently, from the sixth day of storage, a decrease in the green color was observed, leading to browning of the seaweed.

This color is a consequence of the chlorophyll decomposition compounds and even the destruction of the tissue itself, the alteration by enzymatic reactions and most likely microbial activity, as pointed out by Blikra et al. [20]. When UR is stored at 4 °C, degradation and loss of color occurred later, slowing down the effects noted above. However, in both cases, from the second day onwards, color changes began to occur, leading to a slight loss of product quality.

**Figure 2:**
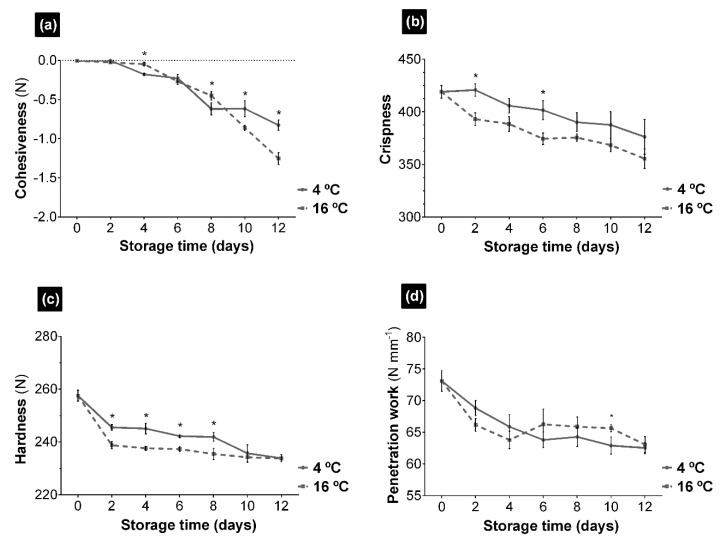
Evolution of *Ulva rigida* (UR) texture parameters during preservation at 4 and 16 °C. (**a**) Cohesiveness parameter in Newton units of force (N); (**b**) Crispness parameter; (**c**) Hardness parameter in Newton units of force (N); (**d**) Penetration work in Newton units of force by millimeter. The asterisk indicates significant differences between preservation temperatures at each sampling point (*p* < 0.05). Data are expressed as mean ± standard deviation (*n* = 3).

According to the texture profile analysis (TPA) of UR (Figure 2), the values of cohesiveness, crispness, hardness and penetration work were affected by the temperature and storage duration, decreasing along time at both temperatures. This effect was also observed in other two seaweeds *Palmaria palmata* and *Gracilaria tikvahiae* [21] and in other marine products, such as sea cucumber [42]. The loss of cohesiveness (Figure 2a), a parameter that determines the degree of compression of a food before it breaks and, therefore, was fractured, occurred at both storage temperatures between day 4 and 6. From day 8 onwards, the greatest differences were observed, as this decreased considerably in seaweed stored at 16 °C.

The degradation of the cell structure, as a result of chemical and enzymatic reactions and even microbial activity, makes the tissue more fragile and, therefore, more easily broken. Likewise, a loss of the crispness, hardness, and penetration work of UR was detected at both temperatures, and although this loss was greater at 16 °C, the differences were not significant. The loss of hardness (Figure 2c), observed from the second day of storage, indicates a decrease in the resistance of the seaweed to compression, possibly related to the withered appearance observed in the samples. In plant cells, turgidity pressure is responsible for their crunchiness, and together with the cell wall and adjacent polysaccharides, contributes to the texture of the plants [47]. The loss of water affects the polysaccharides in the cell wall causing a break down and reducing cell adhesion, resulting in a soft, withered texture, as occurs in vegetables and other seaweed species [21]. The softening of the texture leads to a decrease in the work of penetration, a parameter that indicates consistency or firmness and serves to simulate the mouth bite. This parameter decreased considerably between the first 2 days of storage (Figure 2d). Therefore, TPA showed that changes in the texture of UR occurred in the first days of storage.

### 3.2. Effects of Storage at Different Temperatures on the Chemical Properties of Ulva rigida

Total Volatile Base (TVB) content is one of the most widely used determinations to evaluate the freshness or quality of fish and other marine species [36,38,42]. Both the bacterial activity and the biochemical modifications resulting from the autolytic activity of fish produce a series of basic nitrogenous compounds, such as ammonia, trimethylamine (expressed as trimethylamine nitrogen, TMA-N), dimethylamine (expressed as dimethylamine nitrogen, DMA-N), and monomethylamine, known as total volatile basic nitrogen (TVB-N), which are considered representative of this alteration [48].

**Figure 3:**
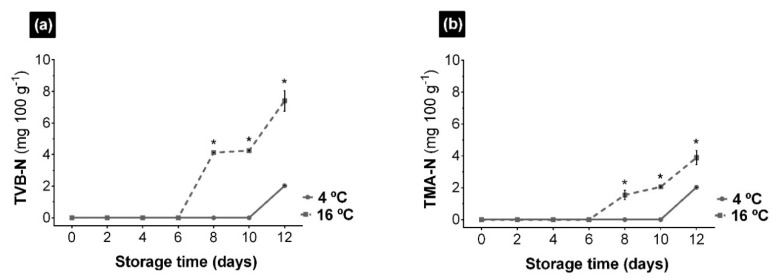
Evolution of Total Volatile Base (TVB-N) (**a**) and Trimethylamine (TMA-N) (**b**) in *Ulva rigida* (UR) during preservation at 4 and 16 °C, expressed in mg 100 g^−1^. The asterisk indicates significant differences between preservation temperatures at each sampling point (*p* < 0.05). Data are expressed as mean ± standard deviation (*n* = 3).

As shown in Figure 3, both the TVB-N and the TMA-N in UR appeared since day 6 in the samples stored at 16 °C and since day 10 in the samples stored at 4 °C. At low temperatures, the levels reached for both parameters were 2 mg 100 g^−1^. Therefore, the TMA-N seemed to be the main component of the TVB-N. In the samples kept at 16 °C, the TMA-N levels after 12 days of storage reached 4 mg 100g^−1^, while the TVB-N levels reached 7 mg 100 g^−1^. In this case the higher values of volatile bases with respect to trimethylamine indicated a hydrolysis of proteins and the appearance of other amines from TMAO, either due to microbial activity or to the endogenous enzymatic activity [49]. The determination of TVB-N had a high correlation with the organoleptic acceptance of fish and is applied in routine tests because it is a relatively simple method [50]. Nevertheless, not all marine species produce volatile bases, and sometimes there is no clear correlation between their content and storage conditions. However, high values in TVB-N are directly related to an advanced state of decomposition [36]. TMA-N is the main component of the TVB-N fraction, a compound produced by the bacterial reduction of trimethylamine oxide (TMAO), considered responsible for the characteristic “unpleasant smell” of fish [51]. TVB-N concentrations did not exceed the limits for the consumption of unprocessed fish products set by the European Community (EC) Commission Regulation No. 1022/2008 [52]. However, the levels found in UR after 12 days of storage were indicative of an advanced state of deterioration and loss of freshness, an aspect to be taken into account when establishing the shelf life of this product.

### 3.3. Effects of Storage at Different Temperatures on the Microbial Cell Count of Ulva rigida

The food industry and health authorities use rapid microbiology tests to verify that the microbiological level of food is safe, which improves decision-making and safety throughout the food chain [53]. In addition, during the storage of fresh products, specific spoilage bacteria proliferate and reduce the shelf life of the product [54]. In addition, microbial growth in foods during storage depends on several intrinsic factors, such as product composition and water content, pH, biological structure, and redox potential [34]. Likewise, external factors, such as temperature, storage time, relative humidity of the environment, composition of the atmosphere or method of preservation [55,56,57]. In general, microbiological tests are expensive, time-consuming and require a lot of manual work. Therefore, it is necessary to find new methodologies to carry out these analyses. In this study, the microbial count by optical microscopy of the exudate liquid was proposed as a rapid and approximate method of microbiological control of the microorganisms responsible for the loss of freshness of UR.

**Figure 4:**
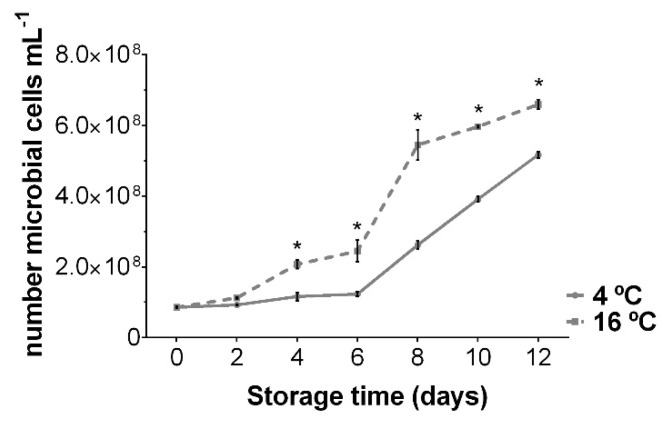
Evolution of the number of microbial cells in an *Ulva rigida* (UR) during its preservation at 4 and 16 °C. The asterisk indicates significant differences between preservation temperatures at each sampling point (*p* < 0.05). Data are expressed as mean ± standard deviation (*n* = 3).

The results of the microbiological analysis performed in UR during its storage at 4 and 16 °C are shown in Figure 4. It was observed that, despite the washing and sanitization treatments carried out on the seaweeds before its packaging, the microbial cell count was high from the beginning, reaching 8.5 × 10^7^ microbial cells mL^−1^. Over the course of the days, this number increased significantly, being more pronounced in the samples stored at 16 °C. Therefore, while at 4 °C the greatest increase occurred on the sixth day, at 16 °C it increased from the second day, reaching final levels of 5.2 × 10^8^ and 6.6 × 10^8^ microbial cells mL^−1^ for each temperature, respectively. Nayyar et al. [21] determined the microbial load of red algae per colony-forming units (CFUs), using a different methodology to the one used in this study, but their evolution over time was similar. Although the microbial count provided a general assessment of microbial activity in the fresh seaweeds, characterization of specific bacteria present in UR is necessary to gain a deeper understanding of microbial spoilage during storage. Compared to the physical and chemical parameters analyzed above, it is clear that microbial growth is not the main cause of loss of quality of stored UR, as is the case with other species [58].

The parameters of TVB-N, TMA-N (Figure 3), and pH (Table 1) are related to the microbial activity causing alteration and deterioration [34,36]. At 4 and 16 °C, an increase in TVB-N and TMA-N was not observed until 10 and 6 days, respectively, in agreement with the pH increase by formation of basic compounds. These results indicated that microbial spoilage and the formation of amines and volatile bases began when the population reached a certain level, 4.0 × 10^8^ and 2.5 × 10^8^ mL^−1^ microbial cells, at 4 and 16 °C, respectively. However, the microorganisms responsible for this deterioration were not the same. At 4 °C, the temperature conditions favored the acidification of the medium and a slight decrease in the a_w_ (Table 1). This allows the growth of bacteria that reduced TMAO and favored the degradation of proteins in the last days of preservation, which led to the generation of TVB-N, TMA-N and an increase in the pH, as in other marine products [36,59]. However, at 16 °C high levels of pH and a_w_ were reached almost since the initial starting point, favoring different microbial development. In the last days, the bacterial populations that caused the drop in pH predominated over those responsible for the formation of TVB-N. This decrease in pH could be due to the increase of acidic compounds, such as acetic acid, hexanoic acid or isovaleric acid, results determined in a previous study on the evolution of volatile compounds of UR during its storage at different temperatures [60]. According to these results, although the determination by count of the number of microbial cells mL^−1^ does not indicate the species that act on UR during its storage, it can be an approximate and fast method to determine the beginning of the symptoms of alteration and degradation that the seaweed suffers and its possible evolution.

### 3.4. Effects of Storage at Different Temperatures on the Sensory Attributes of Ulva rigida

**Figure 5:**
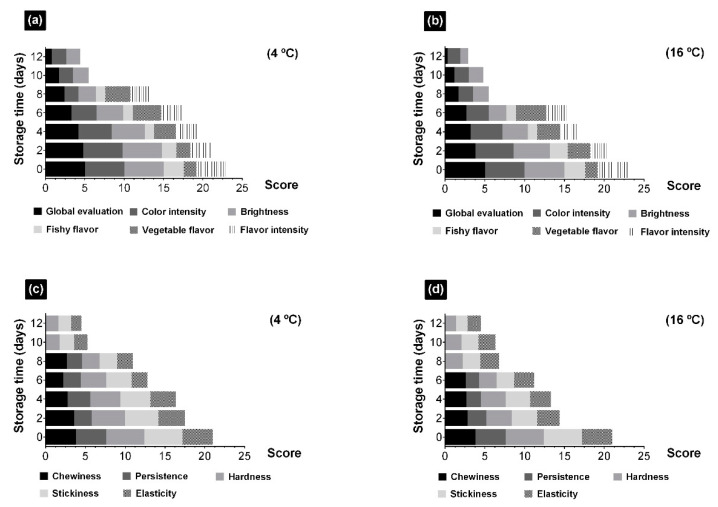
Evolution of appearance and flavor descriptors to global evaluation, color intensity, brightness, fishy flavor, vegetable flavor, and flavor intensity from *Ulva rigida* (UR) stored at 4 (**a**) and 16 °C (**b**) and evolution of tactile mouthfeel and touch descriptors to chewiness, persistence, hardness, stickiness, and elasticity stored at 4 (**c**) and 16 °C (**d**).

Evolution of appearance, flavor, and tactile mouthfeel from UR stored at 4 and 16 °C were evaluated (Figure 5). Ratings of the global evaluation of UR decreased with time, more rapidly and sharply at higher temperatures (Figure 5a,b). This evolution of the appearance during the visual examination was related to a decrease in the color intensity of seaweeds and the loss of brightness, the latter being higher at 16 °C (Figure 5a,b). Since the second day, changes were observed at both storage temperatures, but at 4 °C the overall appearance was preserved for longer. From day 8 onwards, the ratings were below three and one of the most outstanding characteristics of its deterioration was the loss of color uniformity. Color and its uniformity are two of the main characteristics that determine the quality of a fruit or vegetable and is frequently used as its index of freshness, palatability, and nutritional value relating to the intensity of flavor and sweetness [61]. The intense green color and, above all, the brightness, are two of the main characteristics of UR during its visual examination (Figure 5a,b). The loss of brightness occurred progressively from the first days of storage onwards, being more intense in the samples stored at 16 °C. It has been described that the decrease in brightness of fruits and vegetables indicates exceeded development, as it is related to alterations and partial loss of its characteristics of color, flavor, and texture [61,62]. This evolution was also observed in UR and could be promoted by the loss of water by exudate.

In addition to the intensity of color, the intensity of flavor was also lost during storage, being greater at higher temperatures (Figure 5a,b). The characteristic flavors of this species were fish and slightly vegetable. Both flavors and their intensity were modified during storage at both temperatures with slight differences between them. The taste of fish decreased slightly until it disappeared on day 8. At the same time, the vegetable flavor increased and, from day 6 onwards, it began to decrease more significantly at 16 °C.

According to some descriptors related to touch, hardness, stickiness or elasticity, they showed the typical evolution of the decrease in freshness over time as other types of food, such as vegetables, fruits or other marine products (Figure 5c,d). The texture of seaweeds and their flexibility or rigidity (hardness) could be explained by the long chain polysaccharides from cell walls, which gives flexibility and allows adaptation to the type of water currents in which they grow [63,64]. The hardness of UR, considered maximum at the beginning of the monitoring, decreased progressively from the first day onwards and more was more strongly remarked at 16 °C. However, from day 8 onwards no significant differences were observed between both temperatures (Figure 5c,d). The stickiness, in contrast, increased throughout the preservation period from the second day onwards for both temperatures, with greater effect on the samples at 16 °C. The degradation of cell walls, by enzymatic and microbiological activity, as well as of the different components of seaweeds and the release of water during deterioration, led to a loss of texture reflected at a sensory level in a greater softening and increase in stickiness.

**Figure 6:**
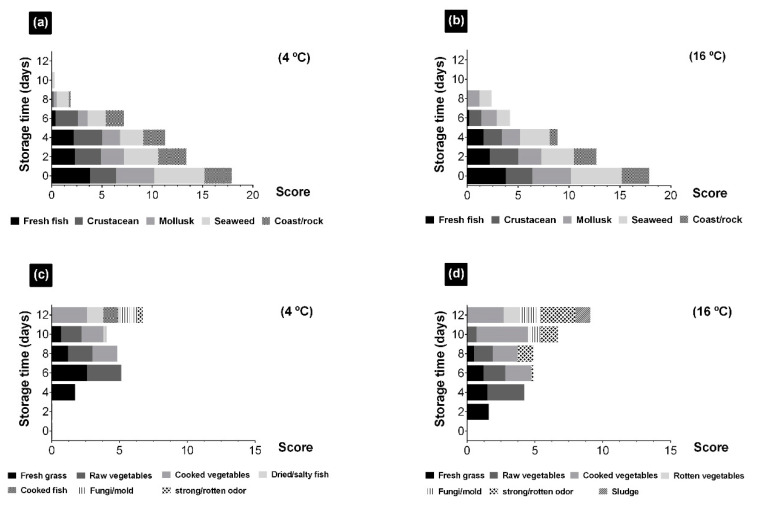
Evolution of positive aromatic descriptors to fresh fish, crustacean, mollusk, seaweed, and coast/rock from *Ulva rigida* (UR) stored at 4 (**a**) and 16 °C (**b**) and evolution of negative aromatic descriptors to fresh grass, raw vegetables, cooked vegetables, rotten vegetables, cooked fish, dried/salted fish, fungi/mold, sludge, and strong/rotten odor stored at 4 (**c**) and 16 °C (**d**).

The aromatic evaluation clearly indicated different effects of temperature and storage time on the seaweeds. At time zero, UR only presented aromas identified as positive and reminiscent of the sea and marine products, such as fish, crustaceans, mollusks, seaweed, and coast/rock (Figure 6a,b). These were clearly in agreement with the initial aromas described in other studies of shelf life evaluation in shellfish [65] and jellyfish [66]. Throughout time, at both storage temperatures, a decrease in overall aromatic intensity was observed. At 4 °C, the characteristic aromas of UR were maintained during the first 4 days, although their intensity gradually decreased. The aroma of fish and of coast/rock disappeared from days 6 and 8 onwards, respectively, and on day 10 the characteristic aromas of quality and freshness of the seaweed disappeared (Figure 6a,b).

At 16 °C, the characteristic aromas of UR were maintained during the first 2 days, but their intensity decreased considerably (Figure 6a,b). From day 4 onwards the aroma of coast/rock was no longer detected, and in the following days the aroma of crustaceans disappeared. From day 10 onwards no characteristic aroma of seaweed was detected. Over time, the positive characteristic aromas of the UR were lost, giving rise to the appearance of other uncharacteristic aromas, both positive and negative (Figure 6c,d). However, the loss or generation of new aromas and, therefore, the aromatic evolution over time depended on the temperature. Parallel to the loss and decrease in intensity of positive aromas, from day 4 onwards for 4 °C, and day 2 for 16 °C, aromas considered as negative were observed (Figure 6c,d). The first days, aromas of fresh grass and raw vegetables were detected, but not rotten odor, that already indicated a significant change in the characteristics of the fresh seaweed (Figure 6a,b). From day 8 onwards for 4 °C, and day 6 for 16 °C, aromas of cooked vegetables appeared. In the last days, fungi/mold, strong/rotten odor aromas were detected, and even sludge and rotten vegetables in the samples at 16 °C, all of which were indicative of deterioration, as a consequence of greater microbial activity at higher temperatures.

The sensory analysis indicated that, although the signs of alteration and deterioration did not start until 8–10 days of storage at 4–16 °C, respectively, the loss of freshness and quality of the seaweed under the conditions studied were observed from the first days of storage onwards. Its sensory characteristics were those that evolved most rapidly, both by enzymatic and microbial activity. These results and those obtained in the physicochemical analyses, could establish the shelf life of UR at one week stored at 4 °C. However, the sensory modifications suffered by the seaweed will condition its use, shortening this period for certain applications, especially culinary.

## 4. Conclusions

The physical, chemical, microbiological, and sensory parameters used in this study indicate a loss of quality and freshness of the seaweed over time, as well as initial symptoms of deterioration. The quality of UR was better preserved at 4 °C, rather than at 16 °C, slowing down both the enzymatic and microbial activity responsible for the deterioration. The pH, the percentage of exudate, as well as the changes in color and texture indicate a loss of seaweed freshness correlated with the increase of microbial cells and the sensory analysis. However, the TVB-N and the TMA-N, are useful chemical parameters to determine the degradation of fresh seaweed by microbial activity and responsible for the sensory characteristics of deterioration during storage, as well as the variation of the pH. Furthermore, although the microbial cell count does not indicate the type of microorganism causing the alterations developed in the medium, it is a quick and simple parameter to determine the microbial load and its possible effect on the seaweeds.

Although the sensory characteristics should remain unchanged during the shelf life of a product, with these results, the shelf life of UR stored at 4 °C can be established at one week. However, after 7 days some modifications on its physical and mainly sensory characteristics are already observed, and can condition its use in culinary applications or development of products from fresh seaweed. In summary, this study provides useful information about the changes in quality and freshness of UR during its storage in refrigeration and the most appropriate methods for its determination and establishment of its shelf life.

## Figures and Tables

**Table 1:** Changes in a_w_, pH and color coordinates of *Ulva rigida* (UR) during storage at 4 and 16 °C.

Property	Storage Temperature (°C)	Storage Time (days)						
		0	2	4	6	8	10	12
**a_w_**	4	0.957 ± 0.002	0.956 ± 0.004 a ^#^	0.953 ± 0.002 a ^#^	0.954 ± 0.001 a	0.951 ± 0.004 a	0.943 ± 0.001 b	0.945 ± 0.002 b
	16		0.950 ± 0.002 b	0.945 ± 0.003 c	0.955 ± 0.003 b	0.946 ± 0.001 c	0.943 ± 0.002 c	0.943 ± 0.001 c
pH	4	6.67 ± 0.07 a	7.24 ± 0.11 b ^#^	7.08 ± 0.08 b	6.49 ± 0.11 c ^#^	6.08 ± 0.05 d ^#^	5.84 ± 0.01 e ^#^	7.24 ± 0.05 b ^#^
	16		6.96 ± 0.09 b	7.19 ± 0.03 c	7.67 ± 0.02 d	7.44 ± 0.04 e	7.06 ± 0.09 b	5.86 ± 0.02 f
Drip loss (%)	4	0.04 ± 0.01 a	0.04 ± 0.01 a	0.43 ± 0.06 b ^#^	2.07 ± 0.06 c ^#^	5.17 ± 0.05 d ^#^	7.63 ± 0.07 e ^#^	7.87 ± 0.06 f ^#^
	16		0.04 ± 0.02 a	0.83 ± 0.05 b	2.82 ± 0.07 c	6.80 ± 0.10 d	8.82 ± 0.04 e	9.23 ± 0.06 f
a*	4	−13.25 ± 0.03 a	−14.54 ± 0.03 b ^#^	−12.73 ± 0.36 a	−14.61 ± 0.19 b ^#^	−14.22 ± 0.35 b ^#^	−15.09 ± 0.03 b ^#^	−12.80 ± 0.11 a ^#^
	16		−11.33 ± 0.37 b	−11.86 ± 0.77 b	−10.26 ± 013 c	−5.65 ± 0.07 d	−11.32 ± 0.41 b	−9.93 ± 0.10 c
b*	4	74.62 ± 2.36 a	78.32 ± 2.12 a ^#^	57.22 ± 1.06 b ^#^	52.61 ± 0.30 c ^#^	49.65 ± 1.04 c ^#^	56.22 ± 1.03 b ^#^	62.39 ± 063 d ^#^
	16		59.09 ± 0.38 b	46.86 ± 0.98 c	40.58 ± 0.94 d	30.83 ± 0.40 e	38.37 ± 1.24 d	36.04 ± 3.89 d
L*	4	66.15 ± 0.62 a	65.30 ± 0.87 a	67.83 ± 0.19 a ^#^	61.40 ± 0.56 b ^#^	60.01 ± 0.34 b ^#^	56.35 ± 0.37 c ^#^	51.39 ± 1.42 d ^#^
	16		66.97 ± 1.13 a	62.19 ± 0.72 b	68.04 ± 0.96 a	67.02 ± 0.65 a	64.87 ± 0.39 a	70.01 ± 1.58 c
*∆E*	4	_	4.29 ± 0.85 a ^#^	17.31 ± 1.05 b ^#^	22.35 ± 1.48 c ^#^	25.53 ± 1.08 c ^#^	20.73 ± 0.93 b ^#^	18.99 ± 1.44 b ^#^
	16		15.51 ± 0.32 a	27.88 ± 0.94 b	34.03 ± 0.95 c	44.26 ± 0.38 d	36.12 ± 1.21 c	38.74 ± 4.00 c

The hash-symbols (#) indicate significant differences between treatments for the same sampling day. Different letters indicate significant differences between consecutive measurement days for the same temperature (*p* < 0.05). Data are expressed as mean ± standard deviation (*n* = 3).

## Data Availability

Data is contained within the article or supplementary material.

## Supplementary Materials

The following are available online at https://www.mdpi.com/2304-8158/10/1/181/s1. Table S1: Sensory analysis sheet prepared for the organoleptic characterization of Ulva rigida (UR).
