# Supplementary material for: Freshness Quality and Shelf Life Evaluation of the Seaweed Ulva rigida through Physical, Chemical, Microbiological, and Sensory Methods
Source: Foods. 2021 Jan 18;10(1):181. doi: 10.3390/foods10010181 (PMC7831315; doi:10.3390/foods10010181)
Supplement: Supplementary file 1 [file foods-10-00181-s001.pdf]

Suppl. Table 1. Sensory analysis sheet prepared for the organoleptic characterization of *Ulva rigida* (UR).

**Sample name:**		**Intensity scale**						**Observations**
**Descriptors**		**0**	**1**	**2**	**3**	**4**	**5**	
Appearance	General aspect							
	Color intensity							
	Brightness							
Marine aromas	Fresh fish							
	Cooked fish							
	Dried/salty fish							
	Rotten fish							
	Crustacean							
	Mollusk							
	Seaweed							
	Coast/rock							
	Sludge							
Vegetable aromas	Fresh grass							
	Raw vegetable							
	Cooked vegetable							
	Rotten vegetable							
	Fruit							
	Fungi/mold							
Other aromas	Spices							
	Urine							
	Strong, rotten odor							
	Other (define)							
Tactile mouthfeel and flavor	Fishy flavor							
	Vegetable flavor							
	Flavor intensity							
	Persistence							
	Chewiness							
Touch	Hardness							
	Stickiness							
	Elasticity							
Global evaluation								
